# The influence of self-reported leisure time physical activity and the body mass index on recovery from persistent back pain among men and women: a population-based cohort study

**DOI:** 10.1186/1471-2458-13-385

**Published:** 2013-04-25

**Authors:** Tony Bohman, Lars Alfredsson, Johan Hallqvist, Eva Vingård, Eva Skillgate

**Affiliations:** 1 Institute of Environmental Medicine, Karolinska Institutet, Box 210, Stockholm SE-17177, Sweden; 2 Department of Public Health Sciences, Division of Public Health Epidemiology, Karolinska Universitetssjukhuset, Stockholm SE-17176, Sweden; 3 Department of Public Health and Caring Sciences, Preventive Medicine, Uppsala University, Box 564, Uppsala SE-75122, Sweden; 4 Department of Medical Science, Occupational and Environmental Medicine, Uppsala University, Akademiska sjukhuset, Uppsala SE-75185, Sweden; 5 Skandinaviska Naprapathögskolan (Scandinavian College of Naprapathic Manual Medicine), Kräftriket 23A, Stockholm SE-11419, Sweden

**Keywords:** Low back pain, Physical activity, Obesity, Epidemiology, Public health

## Abstract

**Background:**

There is limited knowledge about leisure time physical activity and the body mass index (BMI) as prognostic factors for recovery from persistent back pain. The aim of this study was to assess the influence of leisure time physical activity and BMI on recovery from persistent back pain among men and women in a general population.

**Methods:**

The study population (n=1836) in this longitudinal cohort study consisted of participants reporting persistent back pain in the baseline questionnaire in 2002-2003. Data on leisure time physical activity, BMI and potential confounders were also collected at baseline. Information on recovery from persistent back pain (no back pain periods ≥ 7 days during the last 5 years) was obtained from the follow-up questionnaire in 2007. Log-binomial models were applied to calculate Risk Ratios with 95 percent Confidence Intervals (CI) comparing physically active and normal weight groups versus sedentary and overweight groups.

**Results:**

Compared to a sedentary leisure time, all measured levels of leisure time physical activity were associated with a greater chance of recovery from persistent back pain among women. The adjusted Risk Ratios was 1.46 (95% CI: 1.06, 2.01) for low leisure time physical activity, 1.51 (95% CI: 1.02, 2.23) for moderate leisure time physical activity, and 1.67 (95% CI: 1.08, 2.58) for high leisure time physical activity. There were no indications that leisure time physical activity influenced recovery among men, or that BMI was associated with recovery from persistent back pain either among men or among women.

**Conclusions:**

Regular leisure time physical activity seems to improve recovery from persistent back pain among women.

## Background

Back pain (BP) is one of the most common pain conditions worldwide and the prevalence is suggested to increase [1,2]. The proportion of patients reporting BP one year after onset is reported to be as high as 50 to 60 percent [2,3]. These facts support the importance of efforts to find prognostic factors for recovery from BP. To our knowledge there is a lack of evidence concerning such factors as most research have focused on factors associated with bad outcome. A “review of reviews” from 2009 found nine prognostic factors for bad outcome in acute and sub-acute BP consistently reported: older age, poor general health, increased psychological or psychosocial stress, poor relations with colleagues, physically heavy work, functional disability, sciatica, and the presence of compensation [4]. In a contemporary review, no strong evidence was found for any factor to be of prognostic value for persistency of BP [5].

Physical activity may have a positive effect on BP through, for example, increased production of pain inhibiting endorphins and reduction of connective tissue fibrosis which has been suggested to cause BP [6,7]. Overweight, on the other hand, may be negatively associated with back pain through excessive mechanical load, metabolic changes and other biochemical mechanisms [8]. This and the fact that leisure time physical activity and the body mass index (BMI) are modifiable factors and could be an important alternative in self-management of BP emphasize increased knowledge about their prognostic value [9].

Based on seven prospective studies on patients with BP, Hendrick and colleagues found moderate evidence for no association between day-to-day physical activity (occupational, sports and leisure activity) and BP outcomes [10]. The authors urged for continued research on this topic because of few studies and the differences in methodology used. Furthermore, studying bad outcome for BP, Hayden and colleagues found conflicting evidence for BMI to influence the prognosis and no studies about leisure time physical activity as a prognostic factor [4].

There seems to be differences between sexes in several aspects of BP, leisure time physical activity and BMI. Studies report women to have higher prevalence of BP, more severe pain and worse prognosis. Further, women are reported to be more engaged in moderate leisure time physical activity and also to be less prone to have overweight compared to men [2,11-13].

Based on these facts we hypothesized that normal weight and leisure time physical activity would have a positive effect on recovery from persistent back pain, and that this effect may differ between men and women.

In the present study we aimed to investigate the influence of regular leisure time physical activity and BMI on recovery from persistent back pain among men and women in a general population.

## Methods

### Settings and study population

This longitudinal study is based on the Stockholm Public Health Cohort (SPHC, n=23 794). The cohort includes information from two Public Health Surveys performed in Stockholm County, Sweden.

**Figure 1:**
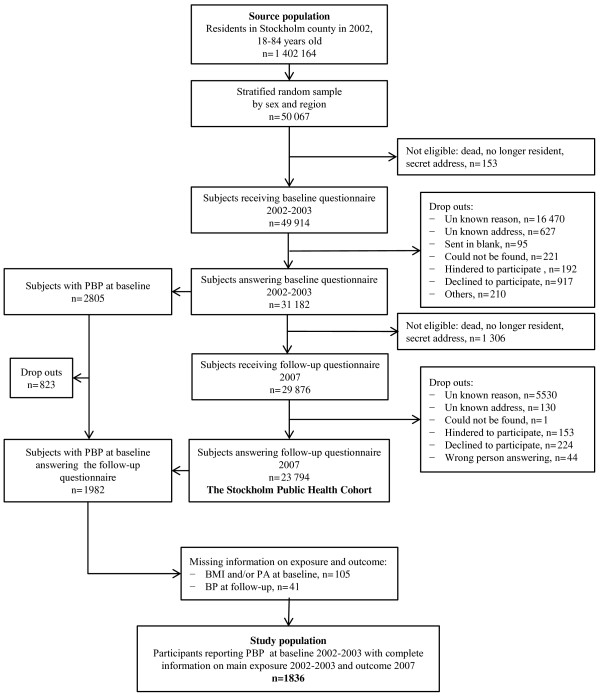
**Flowchart of inclusion process for the study population.** BP: Back pain. PBP: Persistent back pain. BMI: The body mass index. PA: Self-reported regular leisure time physical activity.

The source population was residents, 18 to 84 years old, of Stockholm County, an urban region consisting of 26 municipalities. Selected subjects (n=49 914) received the baseline postal questionnaire between October 2002 and March 2003. The 31 182 subjects responding were sent a follow-up questionnaire between March and August 2007 which 23 794 (76%) subjects completed. Out of those, 1982 subjects reported persistent BP at baseline. After exclusion of 146 subjects with missing data on exposure and outcome the study population included 1836 participants (Figure 1).

Both questionnaires included questions regarding demographic characteristics, physical and psychological health, work related factors, lifestyle and socioeconomics. Data on marital status were retrieved from a Swedish national register [14].

Having persistent back pain (PBP) at baseline was equal to answer “*Yes, every day”* to the question: *“During the previous six months, have you experienced pain in your lower back”?* This question was modified from the Standardized Nordic Questionnaire [15]. The term “persistent” is suggested for back pain present on at least half the days in a 6-months period [16].

### Prognostic factors

Potential prognostic factors were self-reported regular leisure time physical activity (PA) and the body mass index (BMI), reported at baseline.

PA was categorized into four levels using a measure based on the question: *“During the previous 12 months, how physically active have you been during leisure time? If your activity differs between e.g. summer and winter, please estimate the average activity”.* The PA levels were: (a) Sedentary; “*You mostly devote yourself to reading, TV, movies or other sedentary activity during leisure time. You walk, cycle or are active in other ways less than 2 hours a week”,* (b) Low; “*You walk, cycle or are active in other ways at least 2 hours a week, mostly without sweating. Also include walking or cycling to and from work, Sunday walks, ordinary gardening, fishing, table tennis and bowling”*, (c) Moderate; *“You are physically active regularly, 1-2 times a week at least 30 minutes each session with running, swimming, tennis, badminton or other activity that makes you sweat”*, (d) High; *“You devote yourself to e.g. running, swimming, tennis, badminton, aerobic exercise or similar on average at least 3 times a week, each session lasting at least 30 minutes”.*

In a working population with musculoskeletal complaints this PA measure was found to have moderate criterion validity compared to a structural personal interview. Moreover, there were no substantial sex differences in the validity of the measure [17]. Based on the same measure, PA was dichotomized into: Sedentary; alternative a, and Active; alternative b-d.

BMI (kg/m^2^) was calculated using self-reported weight and height and categorized into normal weight (BMI < 25) and overweight (BMI ≥ 25) [18]. The normal weight category included 31 underweight participants (BMI < 18.5) and the overweight category included 270 obese participants (BMI ≥ 30) [18].

### Potential confounders

Information on seventeen potential confounders was gathered at baseline in 2002-2003. These were chosen based on prior research and clinical judgement [4]. Table 1 further describes the potential confounders: smoking habits, alcohol consumption, neck pain, chronic illness, psychological wellbeing, emotional and instrumental social support, socioeconomic class, current occupation, marital status, birthplace, time spent doing housework, physical workload, sick leave and psychosocial work related factors. Most of the questions used to determine the potential confounders have since 1975 regularly been used in previous Swedish public health surveys.

**Table 1:** Potential confounders, their categories and internal drop-outs

**Potential confounder**	**Categories**	**Internal drop-outs **^**a **^**M/W (%)**
**Smoking habits**	Daily smoker, Not daily smoker	0/1
**Alcohol consumption (Grams of 100% alcohol/day)**	No alcohol consumption,	6/5
	Low (males >0 to 40 g/day, females >0 to 20 g/day),	
	Moderate (males >40 to 60 g/day, females >20 to 40 g/day),	
	High (males >60 g/day, females >40 g/day)	
**Neck pain the latest 6 months**	No pain, Two days in total, On average two days a month,	1/1
	On average two days a week, Every day	
**Chronic illness or handicap**	Suffering from long lasting disease, handicap or disability from accidental event? (Yes/No)	2/2
**Psychological wellbeing (GHQ-12) **^**b**^	Reduced psychological wellbeing,	2/1
	Not reduced psychological wellbeing	
**Emotional social support **^**c**^	I have emotional social support,	1/1
	I do not have emotional social support	
**Instrumental social support **^**d**^	I have instrumental social support,	1/1
	I do not have instrumental social support	
**Socioeconomic class (SEI class) **^**e**^	Unskilled and semiskilled workers,	4/5
	Skilled workers,	
	Assistant non-manual employees,	
	Intermediate non-manual employees,	
	Employed/self-employed professionals/ higher civil servants and executives,	
	Self-employed (other than professionals)	
**Current occupation**	Employed, Self-employed, Unemployed,	3/4
	On disability or retirement pension,	
	On leave, Student, “Taking care of the household”	
**Marital status**	Married/registered partnership, Unmarried,	0/0
	Divorced, Widow or widower	
**Birthplace**	Born in Sweden, Born abroad	0/1
**Time spent doing housework a normal weekday**	Almost no time at all, About 30 minutes,	2/1
	1-2 hours, 3-5 hours, > 5 hours	
**Main physical work load the latest 12 months **^**f**^	Sedentary, Light (mobile but no heavy lift),	48/51 ^h^
	Moderate (mobile and some heavy lift), Heavy	
**Sick leave during the latest 12 months**	No sick leave, 1-7 days, > 8 days	38/48 ^h^
**Freedom to decide how to perform work **^**g**^	Never, Mostly not, Mostly, Always	42/51 ^h^
**Freedom to decide what to perform at work **^**g**^	Never, Mostly not, Mostly, Always	43/51 ^h^
**Good collegiality at work **^**g**^	No, Not so good, Quite good, Good	43/51 ^h^

*Note:* Study population (n=1836) with 632 men (M) and 1204 women (W).

^a^ Proportion of missing answer in the potential confounder covariate (%). M = Men, W = women.

^b^ GHQ-12: the twelve-item general health questionnaire. GHQ-12 is regarded as valid to detect cases with reduced psychological wellbeing. Gender, age and education level have no significant effect on the validity [19]. GHQ-12 is described by McDowell [20].

^c^ Emotional social support: Assess whether participants have persons who can support them in handling personal problems or critical life events.

^d^ Instrumental social support: Assess whether participants have persons who can support them in practical matters when they are sick or have practical problems. The social support questions originates from an instrument (ISSI) described by McDowell and tested, as a short form (SS-13), by Undén and Orth-Gomer in a population of Swedish industrial workers [20,21].

^e^ SEI class: Based on occupation and education [22].

^f^ Main physical work load the previous twelve months: For working men and women with musculoskeletal complaints, this question was found to have moderate criterion validity, compared to a structural personal interview (weighted kappa coefficient of 0.68 [95% CI: 0.60-0.76]) [17].

^g^ Freedom to decide how to perform work, Freedom to decide what to perform at work and good collegiality at work: Questions included in the Swedish Demand-Control-Support Questionnaire (DCSQ) [23].

^h^ High proportion of internal dropouts partly due to many participants not working.

We did not treat self-rated health (SRH) as a potential confounder as poor SRH is shown to be a consequence of, rather than the cause for, pain in chronic pain conditions such as BP [24]. If that is true SRH could be regarded as a collider [25]. Baseline SRH was assessed by using an item from the Short Form 36: “In general, would you say your health is?” (very good, good, fair, poor, very poor) [26]. We dichotomized SRH into good (very good, good) and poor (fair, poor, very poor) to be used in additional analyses.

### Outcome

The follow-up questionnaire supplied information about recovery from PBP using a combination of two questions: *“During the last 5-year period have you had back pain for at least 3 consecutive months that has disturbed you considerably?”,* and *“During the last 5-year period have you had back pain, in at least 7 consecutive days but less than 3 consecutive months, that has disturbed you considerably?”* We defined participants as recovered if they answered “*No”* on both questions. Consequently our final definition of recovery from PBP was equal to reporting: *“No periods of considerably disturbing back pain lasting for 7 days or more, during the latest 5-year period”.*

### Statistical methods

#### Main analyses

To study the association between the exposures of interest and recovery from PBP, Risk Ratios (RR) with corresponding 95 percent Confidence Intervals (95% CI) were estimated using log-binomial regression models. We analysed men and women separately. First a crude regression model, including both PA and BMI, were built to analyse whether PA and BMI were associated with recovery from PBP. Potential confounder variables were then, one at a time, added to the crude regression model. If the inclusion changed the crude estimate by 10 percent or more we considered the variable a confounder to be included in the final adjusted model [27,28]. All final analyses were adjusted for age.

#### Additional analyses

We had no information on the intensity of BP at baseline. Therefore, as poor SRH may be a consequence of severe BP, we performed the same adjusted analyses stratified by poor and good SRH using the strata as substitute for more or less severe BP at baseline [24].

The study was approved by the regional ethical review board in Stockholm, Sweden (Diary nr. 2009/457-31). A written informed consent, included in the questionnaires, was obtained from each participant.

Analyses were completed using SAS for Windows version 9.2 TS level 2MO (Cary, NC: SAS Institute).

## Results

The study population (n=1836) had a mean age of 55 years and 66 percent were women. The mean BMI was 26 (SD: 4), and 56 percent of the participants were classified as being overweight. Twenty-three percent of the participants reported sedentary leisure time while low, moderate and high PA was reported by 51, 16 and 10 percent, respectively. Table 2 shows the characteristics for men and women, by level of dichotomized PA and BMI.

**Table 2:** Characteristics of men and women by level of dichotomized main exposure variables (PA and BMI)

	**Men**					**Women**					**Internal drop-out**^**c **^**M/W (%)**
	**All**	**PA **^**a**^		**BMI **^**b**^		**All**	**PA **^**a**^		**BMI **^**b**^		
**Characteristics**		**Sedentary**	**Active**	**Over weight**	**Normal weight**		**Sedentary**	**Active**	**Over weight**	**Normal weight**	
	*(n=632)*	*(n=136)*	*(n=496)*	*(n=416)*	*(n=216)*	*(n=1204)*	*(n=295)*	*(n=909)*	*(n=607)*	*(n=597)*
**Proportion of study population%**	34					66					
**Age, mean (SD)**	56 (14)	56 (15)	56 (14)	56 (13)	54 (16)	55 (15)	57 (16)	55 (15)	58 (14)	53 (16)	0/0
**BMI, mean (SD)**	27 (4)	28 (5)	26 (3)	29 (3)	23 (2)	26 (5)	28 (6)	25 (4)	29 (4)	22 (2)	0/0
**Overweight% **^**d**^	66	68	65	-	-	50	67	45	-	-	0/0
**Sedentary leisure time% **^**e**^	22	-	-	22	20	25	-	-	33	16	0/0
**Daily smoker%**	20	27	18	20	19	20	25	19	19	21	0/1
**Neck pain every day, last 6 months%**	42	53	39	44	39	54	62	52	55	54	1/1
**Low socioeconomic class% **^**f**^	46	50	46	49	42	36	40	35	40	32	4/5
**Born outside Sweden%**	21	34	18	22	19	21	25	20	22	21	0/1
**House work 1 hour or more a day% **^**g**^	48	36	51	45	53	76	69	79	79	73	2/1
**Heavy work% **^**h**^	22	24	22	25	16	13	16	12	14	12	43/51 ^j^
**More than 7 days of sick leave last year%**	32	38	31	34	29	46	56	43	52	41	38/48 ^j^
**Poor self-rated health% **^**i**^	65	82	61	68	59	68	81	64	74	61	2/2

^a^ PA: Self-reported regular leisure time physical activity; Sedentary, mostly sedentary or low intensive activity like walking or cycling < 2 hours a week. Active, low intensive activity like walking or cycling ≥ 2 hours a week or perform more intensive activity ≥ 30 minutes each session in ≥ 1 occasion per week.

^b^ BMI: The body mass index; Overweight, BMI ≥ 25. Normal weight, BMI < 25.

^c^ Proportion of missing answers in question describing the characteristic. Men (M) and women (W).

^d^ Overweight: BMI ≥ 25.

^e^ Sedentary leisure time: Mostly sedentary or low intensive activity like walking or cycling < 2 hours a week.

^f^ Low socioeconomic class: Unskilled and semiskilled workers + skilled workers.

^g^ House work: Taking care of the household a normal weekday, for example cleaning, doing the laundry and cooking.

^h^ Heavy work: Physical work load including frequent heavy lifting.

^i^ Fair, poor or very poor self-rated health.

^j^ High proportion of internal dropouts partly due to many participants not working.

### Main results

At the follow-up in 2007, 21 percent of the participants had recovered from PBP. Table 3 presents results from the log-binomial regression analyses, crude and adjusted, for the associations between exposures and outcome. Neck pain was the only variable found to be a confounder and only among men.

**Table 3:** Crude and adjusted RR of recovery from PBP associated with levels of PA and BMI

**Exposure**	**Men**				**Women**			
	***(n=632)***				***(n=1204)***			
	**Recovered **^**a**^	**Not recovered**	**Crude**^**b**^	**Adjusted**^**c**^	**Recovered **^**a**^	**Not recovered**	**Crude **^**b**^	**Adjusted **^**c**^
	***(n=142, 22%)***	***(n=490, 78%)***	**RR**	**RR**	***(n=245, 20%)***	***(n=959, 80%)***	**RR**	**RR**
	***n (%)***	***n (%)***	**(95% CI)**	**(95% CI)**	***n (%)***	***n (%)***	**(95% CI)**	**(95% CI)**
**PA **^**d**^								
Sedentary ^e, j^	30 (22)	106 (78)	Ref.	Ref.	43 (15)	252 (85)	Ref.	Ref.
Low ^f^	72 (22)	253 (78)	1.01	0.92	132 (22)	477 (78)	1.46	1.46
			(0.69, 1.47)	(0.64, 1.32)			(1.06, 2.01)	(1.06, 2.01)
Moderate ^g^	24 (24)	77 (76)	1.06	0.97	43 (23)	148 (77)	1.50	1.51
			(0.66, 1.71)	(0.61, 1.56)			(1.02, 2.21)	(1.02, 2.23)
High ^h^	16 (23)	54 (77)	1.03	0.91	27 (25)	82 (75)	1.66	1.67
			(0.60, 1.75)	(0.54, 1.54)			(1.07, 2.55)	(1.08, 2.58)
**BMI **^**i**^								
Overweight ^j^	91 (22)	325 (78)	Ref.	Ref.	114 (19)	493 (81)	Ref.	Ref.
Normal weight	51 (24)	165 (76)	1.07	1.03	131 (22)	466 (78)	1.09	1.10
			(0.79, 1.45)	(0.77, 1.39)			(0.87, 1.37)	(0.87, 1.38)

*Note:* Risk ratio (RR) together with corresponding 95% confidence interval (95% CI). Men and women are reported separately.

^a^ Recovered from persistent back pain (PBP) = “No periods of considerably disturbing back pain lasting for 7 days or more, during the latest 5-year period”. Reported at follow-up in 2007.

^b^ Crude log-binomial regression model including PA and BMI.

^c^ Adjusted log-binomial regression model including PA, BMI, age and neck pain for men and PA, BMI and age for women.

^d^ PA: Self-reported regular leisure time physical activity the latest 12 months. Reported at baseline in 2002.

^e^ Sedentary: Mostly sedentary or low intensive activity like walking or cycling < 2 hours a week.

^f^ Low: Low intensive activity like walking or cycling ≥ 2 hours a week.

^g^ Moderate: At least 30 min activity, 1-2 sessions per week with activities like running, swimming, tennis, aerobics or similar.

^h^ High: At least 30 min activity, ≥ 3 sessions per week with activities like running, swimming, tennis, aerobics or similar.

^i^ BMI: Overweight; BMI ≥ 25, Normal weight; BMI < 25. Reported at baseline in 2002.

^j^ Reference category (RR = 1.0).

Compared to sedentary leisure time, the chance of recovery from PBP was greater for women that were physically active during leisure time. The adjusted RR among women was 1.46 (95% CI: 1.06, 2.01) for low PA, 1.51 (95% CI: 1.02, 2.23) for moderate PA, and 1.67 (95% CI: 1.08, 2.58) for high PA. No analyses indicated that PA was associated with recovery from PBP among men or that BMI was associated with recovery from PBP, either among men or among women.

### Additional results

Table 4 shows the adjusted RR of recovery from PBP associated with PA and BMI, respectively, in men and women stratified by good and poor self-rated health (SRH). The RR´s for the association between levels of PA and recovery from PBP were well above 1.0, though not statistically significant, both for women with good SRH (RR´s: 1.35-1.66) and women with poor SRH (RR´s: 1.34-1.77).

**Table 4:** Adjusted RR of recovery from PBP^* ^**associated with levels of main exposures by sex and SRH**

**Self-rated health (SRH) **^**a**^								
**Exposure**	**Men *(n=621) ***^***b***^				**Women *(n=1176) ***^***b***^			
		**Good SRH **^**c**^		**Poor SRH **^**c**^		**Good SRH **^**c**^		**Poor SRH **^**c**^
		*(n=217, 35%)*		*(n=404, 65%)*		*(n=377, 32%)*		*(n=799, 68%)*
	*n*	RR (95% CI)	*n*	RR (95% CI)	*n*	RR (95% CI)	*n*	RR (95% CI)
**PA **^**d**^								
Sedentary ^e, j^	24	Ref.	108	Ref.	55	Ref.	232	Ref.
Low ^f^	107	0.82 (0.41, 1.62)	213	0.77 (0.49, 1.22)	191	1.66 (0.91, 3.03)	403	1.36 (0.91, 2.03)
Moderate ^g^	48	0.81 (0.38, 1.75)	52	0.92 (0.49, 1.72)	79	1.65 (0.86, 3.18)	109	1.34 (0.78, 2.30)
High ^h^	38	0.74 (0.33, 1.64)	31	0.76 (0.32, 1.80)	52	1.35 (0.65, 2.83)	55	1.77 (0.96, 3.27)
**BMI **^**i**^								
Overweight ^j^	130	Ref.	278	Ref.	152	Ref.	441	Ref.
Normal weight	87	1.24 (0.84, 1.85)	126	0.77 (0.49, 1.22)	225	1.07 (0.76, 1.50)	358	1.13 (0.82, 1.56)

*Note:* Risk ratio (RR) together with corresponding 95% confidence interval (95% CI). Men and women are reported separately and presented by good and poor self-rated health.

^*^ Recovered from persistent back pain (PBP) = “No periods of considerably disturbing back pain lasting for 7 days or more, during the latest 5-year period”. Reported at follow-up in 2007.

^a^ Good = very good and good self-rated health; Poor = fair, poor and very poor self-rated health.

^b^ Number of participants was less than the study population according to missing values in self-rated health. Eleven men and 28 women had missing values in self-rated health.

^c^ Adjusted log-binomial regression model including PA, BMI, age and neck pain for men and PA, BMI and age for women.

^d^ PA: Self-reported regular leisure time physical activity the latest 12 months. Reported at baseline in 2002.

^e^ Sedentary: Mostly sedentary or low intensive activity like walking or cycling < 2 hours a week.

^f^ Low: Low intensive activity like walking or cycling ≥ 2 hours a week.

^g^ Moderate: At least 30 min activity, 1-2 sessions per week with activities like running, swimming, tennis, aerobics or similar.

^h^ High: At least 30 min activity, ≥ 3 sessions per week with activities like running, swimming, tennis, aerobics or similar.

^i^ BMI: Overweight; BMI ≥ 25, Normal weight; BMI < 25. Reported at baseline in 2002.

^j^ Reference category (RR = 1.00).

## Discussion

In this study we found that a non-sedentary leisure time improved recovery from persistent back pain (PBP) among women in a general population. There were no indications that regular leisure time physical activity (PA) influenced recovery for men, or that recovery from PBP was associated with BMI either among men or among women. Furthermore, the additional analyses indicated that PA may have a positive effect on recovery for women no matter the severity of the PBP.

### Comparison with other studies

We found only few studies with an aim similar to ours. However, these studies have differences concerning design, outcome, study sample as well as definition of disease which may explain the somewhat conflicting results [4]. In a community-based study with subjects reporting BP the previous month, neither leisure activity nor sports were found to be predictive for BP one year later [29]. Hurwitz and colleagues found increased levels of recreational physical activity to reduce pain and disability among BP patients randomized to chiropractic or medical care [6]. In contrast, Mortimer and colleagues found no effect from recreational exercise on pain and disability among men or among women, five years after seeking care for BP [30]. A cohort study, following sick-listed BP patient’s showed no or moderate physical activity to be associated with worse disability and pain at one-year follow-up [31]. The authors observed no relation between BMI and disability or pain. Further, a review of prognostic factors for patients sick-listed with acute BP found moderate evidence for BMI and no evidence for physical fitness or sports to be prognostic factors for duration of sick leave [13].

In our study only 21 percent of the participants recovered from BP when other studies report recovery proportions of 50 to 60 percent [2,4]. This discrepancy may reflect the recurrent pattern characteristic for BP as we defined recovery to be free of pain for five years and the other studies reported recovery one year after onset [32]. Despite our stringent definition of recovery we found a positive effect of PA among women, supporting its importance as a prognostic factor.

Why would PA have a positive effect on recovery from PBP in general and why among women only? These mechanisms have not yet been fully elucidated and we can only speculate. PA could for example; (1) give general increase in circulation and production of endorphins suggested to reduce back pain [6], (2) reverse connective tissue fibrosis and neurally-mediated inflammation probably linked to back pain [7], (3) be of benefit in rheumatoid arthritis, fibromyalgia and osteoporosis, health problems possibly resulting in back pain and more common among women [33,34] and (4) reduce pain more among women than men as indicated by experimental studies [35].

### Strengths and limitations

We believe that our study has some strengths to mention. First, the longitudinal study design supports a causal relationship between PA and recovery from PBP among women [27]. Second, the large number of potential confounders assessed strengthens the internal validity, although we cannot rule out residual or unmeasured confounding. Third, we consider our overall sample size to be large for a study concerning every day BP for six months or more. Still the analyses of some exposure categories (e.g. 27 recovered women with high PA) were somewhat hampered by lack of statistical power and should therefore be interpreted with caution. Fourth, the baseline definition of PBP indicates back pain severe enough to have negative consequences for the affected individual as well as for the society and therefore important to study.

One problem with studies concerning BP is the lack of consensus for the definition of BP episodes as well as definition for recovery from BP [36,37]. In a Delphi study from 2011 aiming to standardize the definition of recurrent BP the definition for recovery was “at least 30 days pain-free” [38]. This definition was also incorporated in the definition of an episode of low back pain suggested by de Vet in 2002 [39]. To our knowledge, this is as close to a consensus on a definition of recovery from BP that there is today.

We believe that our recovery outcome (*“No periods of considerably disturbing back pain lasting for 7 days or more, during the latest 5-year period”*) might be even more relevant. First it incorporates not only pain but also disability which is recognized to be clinically important when defining BP and recovery from BP [36]. Second, it accounts for the fact that BP is a recurrent disorder and therefore being free of disabling pain for 5 years is a very stringent definition.

The present study also has limitations. We had no baseline information on pain intensity or back pain prior to inclusion into the cohort why we could not test these factors as potential confounders. If women with more severe PBP had lower baseline levels of PA than women with less severe PBP, bias due to reversed causation may be present [27,40]. Then our results may have been overestimated. However this effect may be counterbalanced if some women with more severe PBP were more active than women with less severe PBP. The later could be a result of advice to stay active given by health care providers. Furthermore, the additional analyses stratified on self-rated health (as a substitute for severity of BP) indicated that being physically active is beneficial for women no matter the severity of the PBP. There may also be a problem with unmeasured confounding from health care utilization.

When using self-reported BMI, overweight participants may be classified as having normal weight [41]. Such misclassification would be non-differential and tend to dilute the strength of association [27]. This could explain the lack of association with recovery from BP in our study. Nevertheless, BMI is widely used and recommended as a baseline exposure measurement in cohort studies with BP as outcome [42]. Non-differential misclassification may also concern the measurements of PA with a dilution of potential associations as a consequence. If men tend to misclassify their PA to a higher degree than women, non-differential misclassification may partly explain the different findings between the sexes. However, our measurement method for PA has been reported to be useful for categorizing adults into different levels of PA, based on the physical activity in the different groups as measured by accelerometer [43].

Due to the five-year recall period recovery from PBP could have been misclassified [27]. If for example physically active participants remember their periods of back pain better as it may interfere with their activities, misclassification could either exaggerate or underestimate a true effect. We regard the risk for such differential misclassification as small but if present it would most probably underestimate the effect size.

The follow-up definition of PBP incorporates a dimension of disability different from the baseline definition of PBP. This may cause differential misclassification of outcome if active participants found BP more disabling because it prevents their PA. Again the most probable consequence would be dilution of the estimated association.

Attrition during the sampling process of the cohort and the fact that we select a subsample from the general population could bias our findings and affect external validity. However, some facts contradict this. First, public health survey data from extensive questionnaires with participants blinded to the study hypothesis supports missing at random [44]. Second, given the associations studied are causal we believe that our results are valid also for subjects that dropped out or were excluded.

We believe in summary, considering the strengths and limitations that the associations found in this study are valid.

## Conclusions

To our knowledge this is the first study assessing the sex specific associations between the exposures, leisure time physical activity and BMI, and recovery from persistent back pain (PBP). Our findings indicate that regular leisure time physical activity may improve recovery from PBP among women while there seems to be no associations between BMI and recovery from PBP. This may challenge the common beliefs that overweight affects the prognosis of PBP even though further research is needed. Finally, based on our results, we consider it important that future research on prognostic factors for back pain addresses men and women separately otherwise sex differences may be hidden.

## Competing interests

The authors declare that they have no competing interests.

## Authors’ contributions

All authors contributed to the design of the study and interpretation of the data. TB made the statistical analyses and wrote the first manuscript version. All authors critically revised all versions of the manuscript and finally approved the last version.

## Pre-publication history

The pre-publication history for this paper can be accessed here:

http://www.biomedcentral.com/1471-2458/13/385/prepub
